# Complete Genome Sequence of *Pseudomonas chlororaphis* subsp. *aurantiaca* Reveals a Triplicate Quorum-Sensing Mechanism for Regulation of Phenazine Production

**DOI:** 10.1264/jsme2.ME16162

**Published:** 2017-02-25

**Authors:** Tomohiro Morohoshi, Takahito Yamaguchi, Xiaonan Xie, Wen-zhao Wang, Kasumi Takeuchi, Nobutaka Someya

**Affiliations:** 1 Department of Material and Environmental Chemistry, Graduate School of Engineering, Utsunomiya University7–1–2 Yoto, Utsunomiya, Tochigi 321–8585Japan; 2 Center for Bioscience Research and Education, Utsunomiya University350 Mine-machi, Utsunomiya, Tochigi 321–8505Japan; 3 Key Laboratory for Eco-Efficient Polysilicate Materials, School of Environment and Energy, Peking University Shenzhen Graduate SchoolGuangdong 518055China; 4 Division of Plant and Microbial Sciences, Institute of Agrobiological Sciences, National Agriculture and Food Research Organization2–1–2 Kannondai, Tsukuba, Ibaraki 305–8602Japan; 5 Institute of Vegetable and Floriculture Sciences, National Agriculture and Food Research Organization3–1–1 Kannondai, Tsukuba, Ibaraki 305–8519Japan

**Keywords:** *Pseudomonas chlororaphis*, quorum sensing, acylhomoserine lactone, phenazine, biocontrol

## Abstract

*Pseudomonas chlororaphis* subsp. *aurantiaca* StFRB508 regulates phenazine production through *N*-acyl-l-homoserine lactone (AHL)-mediated quorum sensing. Two sets of AHL-synthase and AHL-receptor genes, *phzI*/*phzR* and *aurI*/*aurR*, have been identified from the incomplete draft genome of StFRB508. In the present study, the complete genome of StFRB508, comprising a single chromosome of 6,997,933 bp, was sequenced. The complete genome sequence revealed the presence of a third quorum-sensing gene set, designated as *csaI*/*csaR*. An LC-MS/MS analysis revealed that StFRB508 produced six types of AHLs, with the most important AHL being *N*-(3-hydroxyhexanoyl)-l-homoserine lactone (3-OH-C6-HSL). PhzI mainly catalyzed the biosynthesis of 3-OH-C6-HSL, while AurI and CsaI catalyzed that of *N*-hexanoyl-l-homoserine lactone and *N*-(3-oxohexanoyl)-l-homoserine lactone, respectively. A mutation in *phzI* decreased phenazine production, whereas that in *aurI* or *csaI* did not. A *phzI aurI csaI* triple mutant (508ΔPACI) did not produce phenazine. Phenazine production by 508ΔPACI was stimulated by exogenous AHLs and 3-OH-C6-HSL exerted the strongest effects on phenazine production at the lowest concentration tested (0.1 μM). The plant protection efficacy of 508ΔPACI against an oomycete pathogen was lower than that of wild-type StFRB508. These results demonstrate that the triplicate quorum-sensing system plays an important role in phenazine production by and the biocontrol activity of StFRB508.

Phenazine and its derivatives are orange-pigmented heterocyclic products of bacterial secondary metabolism, and are known for their broad-spectrum antifungal activity (12, 18). A wide variety of phenazine derivatives, such as phenazine-1-carboxylic acid (PCA) and phenazine-1-carboxamide (PCN), are produced by various Gram-negative and Gram-positive genera, including *Brevibacterium*, *Burkholderia*, *Pseudomonas*, and *Streptomyces* (18). The core phenazine biosynthetic gene cluster contains seven genes (*phzABCDEFG*) and is present in several *Pseudomonas* species such as *Pseudomonas chlororaphis*, *P. aeruginosa*, and *P. fluorescens* (12). Phenazine-producing *Pseudomonas* species have been studied as biocontrol agents against many plant diseases (27). *P. chlororaphis* PCL1391 produces PCN and controls tomato foot and root rot caused by *Fusarium oxysporum* (3). PCN and PCA produced by *P. aeruginosa* PNA1 are involved in the biocontrol of *Pythium myriotylum* on cocoyam (24).

Quorum sensing is a bacterial cell-cell communication process and modulates cell density-dependent gene expression (16). *N*-Acyl-l-homoserine lactone (AHL) has been identified as a signaling compound involved in quorum-sensing in many Gram-negative bacteria (16). The LuxI/LuxR system is used by many Gram-negative bacteria to control the expression of genes regulated by quorum sensing (16). Members of the LuxI protein family catalyze AHL biosynthesis. When AHL concentrations reach a threshold due to increases in bacterial cell density, members of the LuxR protein family, comprising AHL receptors, bind to AHL, thereby regulating the expression of many genes. Previous studies reported that the production of some antimicrobial compounds, such as carbapenem, prodigiosin, and pyrrolnitrin, is regulated by AHL-mediated quorum sensing (21, 25). The expression of the phenazine biosynthesis gene cluster in fluorescent pseudomonads is activated in the stationary growth phase in a manner that depends on the production of AHLs (17). The quorum sensing-mediated regulation of the phenazine biosynthesis gene cluster in some *P. chlororaphis* strains has been investigated in detail (3, 7, 27). AHLs are mainly synthesized by PhzI in phenazine-producing *P. chlororaphis* (27). The AHL receptor protein, PhzR, binds to AHLs and activates the expression of the phenazine biosynthetic gene cluster. Another quorum-sensing system, *csaI*/*csaR*, was detected in *P. chlororaphis* subsp. *aureofaciens* 30–84, but was not involved in the regulation of the phenazine biosynthetic gene cluster (27). We previously isolated *P. chlororaphis* subsp. *aurantiaca* StFRB508 from potato roots and identified two functional quorum-sensing systems, *phzI*/*phzR* and *aurI*/*aurR*, in the incomplete draft genome sequence of StFRB508 (13). The findings of a thin-layer chromatography (TLC) analysis revealed that PhzI catalyzes the biosynthesis of *N*-(3-hydroxyhexyanoyl)-l-homoserine lactone (3-OH-C6-HSL), while AurI catalyzes that of *N*-butanoyl-l-homoserine lactone (C4-HSL) and *N*-hexanoyl-l-homoserine lactone (C6-HSL). Phenazine production in AHL-negative mutants was stimulated by exogenous AHLs and the most active AHL was 3-OH-C6-HSL. In an attempt to elucidate the quorum-sensing system involved in the regulation of phenazine production in the present study, the complete genome of StFRB508 was sequenced and a third quorum-sensing system was identified. In addition, the concentrations and structures of AHLs produced by StFRB508 were accurately measured and elucidated using LC-MS/MS. We also investigated the biocontrol activity of StFRB508 and its quorum-sensing-deficient mutant.

## Materials and Methods

### Bacterial strains, compounds, and growth conditions

The bacterial strains used in this study are listed in Table S1. All bacterial strains were grown in Luria-Bertani (LB) medium (10 g L^−1^ peptone, 5 g L^−1^ yeast extract, and 5 g L^−1^ NaCl). Solid bacterial media were prepared by the addition of agar at a final concentration of 1.5%. Antibiotics were added as required at final concentrations of 100 μg mL^−1^ carbenicillin and 50 μg mL^−1^ kanamycin. The AHLs used in this study, C6-HSL, *N*-(3-oxohexanoyl)-l-homoserine lactone (3-oxo-C6-HSL), *N*-(3-oxooctanoyl)-l-homoserine lactone (3-oxo-C8-HSL), *N*-(3-oxodecanoyl)-l-homoserine lactone (3-oxo-C10-HSL), and *N*-(3-oxododecanoyl)-l-homoserine lactone (3-oxo-C12-HSL), were synthesized using a previously described method (2). 3-OH-C6-HSL, *N*-(3-hydroxyoctanoyl)-l-homoserine lactone (3-OH-C8-HSL), *N*-(3-hydroxydecanoyl)-l-homoserine lactone (3-OH-C10-HSL), and *N*-(3-hydroxydodecanoyl)-l-homoserine lactone (3-OH-C12-HSL) were prepared from 3-oxo-C6-HSL, 3-oxo-C8-HSL, 3-oxo-C10-HSL, and 3-oxo-C12-HSL, respectively, via the reduction of the ketone functional group with NaBH_4_ by a previously described method (19).

### Genome sequencing of StFRB508

The single- and paired-end whole-genome shotgun sequencing of StFRB508 was performed using a Roche Genome Sequencer FLX Titanium pyrosequencer (Eurofins Genomics, Tokyo, Japan) as described in a previous study (13). Gap closure was attempted using gap-spanning clones and PCR products. The prediction of putative coding sequences and gene annotation were performed using the Microbial Genome Annotation Pipeline (http://www.migap.org/). Briefly, protein-coding sequences (CDSs) were predicted by the combined use of MetaGeneAnnotator (14), RNAmmer (8), tRNAScan (11), and BLAST (1).

### Cloning and disruption of quorum-sensing genes of StFRB508

The *phzIR*, *aurIR*, and *csaIR* coding regions on the StFRB508 genome were amplified with Blend Taq plus DNA polymerase (Toyobo, Osaka, Japan) and specific primers (Table S2). PCR reaction conditions were as follows: 27 cycles at 94°C for 30 s, 60°C for 30 s, and 74°C for 2 min. PCR products were cloned into the pGEM-T easy cloning vector (Promega, Tokyo, Japan). In order to remove the internal sequence of the target genes, sequences upstream and downstream of the target gene were amplified using pGEM-T easy containing constructed plasmids as the template with specific primers for gene deletion (Table S2). The amplified PCR fragments were excised by *Hin*dIII digestion and self-ligated. The gene-coding region with a deletion in an internal sequence was excised by *Eco*RI and inserted into the *Eco*RI site of the suicide vector, pK18mobsacB (20), in order to create the plasmids for gene deletion. Gene-deletion plasmids were conjugated from *Escherichia coli* S17-1 λ*pir* (22) using StFRB508 mutants as recipients. The StFRB508 recombinants corresponding to single-crossover events were selected on LB agar containing kanamycin and carbenicillin. Deletion mutants of the quorum-sensing genes in StFRB508 were generated by homologous recombination with *sacB* selection. The single-crossover mutants of StFRB508 were streaked onto an LB agar plate with 10% (w/v) sucrose in order to select homologous recombinants. The presence of the expected internal gene deletion in StFRB508 was confirmed by PCR.

### Assessment of phenazine production

In the agar plate assay, StFRB508 and its mutants were grown on LB agar plates with or without 1% (w/v) monosaccharides such as d-glucose, d-fructose, d-galactose, l-arabinose, and d-xylose (Kanto Chemical, Tokyo, Japan). A sterile toothpick was dipped into a bacterial suspension and inoculated onto new LB agar prepared in 24-well plates. After an incubation at 30°C for 2 d, phenazine production was identified by the presence of orange pigments. Phenazine production was categorized into three levels: (i) high production (deep orange with diffusion), (ii) low production (moderate orange without diffusion), and (iii) no production (white or very pale yellow).

Phenazine production was also evaluated by the broth assay as described previously with modifications (13). StFRB508 and its mutants were grown for 15 h, inoculated into fresh LB medium (1% inoculum) with or without AHLs, and further incubated for 18 h. One milliliter of each culture was placed in a 1.5-mL microtube. After centrifugation for 5 min, the concentration of phenazine in the culture supernatant was evaluated by measuring absorbance at 365 nm (A_365_). The turbidity of the culture suspension was assessed by measuring optical density at 600 nm (OD_600_). Phenazine production was evaluated as relative production (A_365_/OD_600_), the maximum value of which equaled 100%.

### Extraction and identification of AHLs

StFRB508 and its mutants were inoculated into 4 mL of LB medium containing 1% (w/v) glucose and incubated at 30°C for 18 h under shaking conditions at 150 rpm. Full-grown cultures (500 μL) were transferred into 50 mL fresh medium and incubated at 30°C for 24 h. Cells were removed by centrifugation at 10,000×*g* for 5 min. Culture supernatants were concentrated by evaporation using a rotary evaporator. They were then extracted with a 3-fold volume of ethyl acetate in a separation funnel. The extract was evaporated to dryness using a rotary evaporator and then dissolved in 500 μL dimethylsulfoxide. The chemical structures of extracted AHLs were analyzed by Liquid Chromatography-Tandem Mass Spectrometry (LC-MS/MS) as described previously (15).

### Quantification of AHLs by LC-MS/MS

The quantification of AHLs was performed using a triple quadrupole/linear ion trap instrument (LIT) (QTRAP5500; AB SCIEX, Tokyo, Japan) with an electrospray ionization (ESI) source coupled to a UHPLC system (Nexera X2; Shimadzu, Kyoto, Japan) in the multiple reaction monitoring (MRM) analysis mode. Chromatographic separation and MS/MS spectra were recorded as reported previously (15). An MRM analysis was performed at 15 V collision energies for the transitions of *m*/*z* 200→102 for C6-HSL, 214→102 for 3-oxo-C6-HSL, 216→102 for 3-OH-C6-HSL, 244→102 for 3-OH-C8-HSL, 272→102 for 3-OH-C10-HSL, and 300→102 for 3-OH-C12-HSL. Data acquisition and analyses were performed with Multi Quant software ver. 3.0.1 (AB SCIEX). The quantification of AHLs was conducted using synthetic standards. Exudate samples were dissolved in 50% aqueous acetonitrile and filtered through a spin column (Ultrafree-MC 0.45 μm filter unit; Millipore, Bedford, MA). An aliquot of filtered 50% aqueous acetonitrile sample solutions was diluted with a volume of either pure 50% acetonitrile or 50% acetonitrile containing known amounts of the AHL standards. The increase in the peak area on the chromatogram corresponded to the amounts of the AHL standards added, thereby enabling the amounts of AHLs in the samples to be estimated. Results were reproduced in three repeated experiments.

### Plant disease suppression assays

The biocontrol activity of each strain was evaluated as described previously (23), with slight modifications. Flasks containing 20 g of a 2:1 mixture of vermiculite and peat moss were planted with three cucumber seedlings each and treated with *Pythium ultimum* MAFF425494. StFRB508 mutants were added to soil as a suspension (4 mL in each flask) of cells washed twice in sterile distilled water to give 2×10^7^ CFU g^−1^ soil. Control flasks received the same amount of sterile water. Seedlings were covered with 5 g of untreated soil and flasks were sealed with aerated silicon caps. Microcosms were incubated in a growth chamber at 60% relative humidity and 26°C with light for 16 h, followed by an 8-h dark period. No watering was necessary. After a 7-d incubation, biocontrol activity was assessed by counting the surviving plants in each flask. Data in Table 1 represent means from two individual repetitions of the same experiment. Data from both experiments were analyzed for a trial by a treatment interaction using an analysis of variance, which indicated that data from the two independent trials may be pooled. Means were separated using Tukey’s honest significant difference test (at P≤0.05). Statistical analyses were performed using R version 3.0.1 (http://www.r-project.org/).

### Nucleotide sequence accession number

The complete genome sequence of *P. chlororaphis* subsp. *aurantiaca* StFRB508 has been deposited in the DDBJ/ENA/GenBank databases under accession no. AP014623.

## Results and Discussion

### Complete genome sequencing of StFRB508

We previously reported the incomplete draft genome sequence of StFRB508 (13). Two pairs of *luxI* and *luxR* homologues, designated as *phzIR* and *aurIR*, were detected in the draft genome of StFRB508. However, the draft genome sequence of StFRB508 still contains numerous long sequencing gaps. In order to obtain the complete genome sequence of StFRB508, 896,121 paired-end reads with an average read length of 154 bases were re-assembled by applying Eurofins *in silico* Gap Closure pipeline with manual inspection. Consequently, reads were assembled into 34 large contigs (>2,000 bp). The results of PCR for closing assembly gaps revealed that the complete genome of StFRB508 comprised a single circular chromosome of 6,997,933 bp with an average G+C content of 62.82% (Fig. S1). The genome contained 6,309 protein-coding, 16 rRNA, and 67 tRNA genes. In order to elucidate complete quorum-sensing gene networks, we searched for additional AHL-synthase gene homologues in the complete genome. One predicted an ORF (PCAU_2446), which encoded 219 amino acids, showing high identity (more than 94%) to the AHL synthase CsaI from *P. chlororaphis* subsp. *aureofaciens* 30–84 (27). A putative *luxR* homologous gene, designated as *csaR* (PCAU_2447), was mapped upstream of *csaI* as well as 30–84. Furthermore, a previous study reported that HdtS from *Pseudomonas fluorescens* F113 is a third protein family capable of AHL biosynthesis (9). The *hdtS* homologue was also detected in the complete genome of StFRB508 (PCAU_6164). The complete genome sequence of another *P. chlororaphis* subsp. *aurantiaca* strain JD37 has also been described (6). Therefore, we searched quorum sensing-related genes in the complete genome of JD37. Based on the results obtained, *phzIR*, *aurIR*, *csaIR*, and *hdtS* genes were found to be shared between StFRB508 and JD37. These results demonstrate that these quorum-sensing genes are conserved in *P. chlororaphis* subsp. *aurantiaca*.

### Quorum sensing-regulated phenazine biosynthesis in StFRB508

**Fig. 1:**
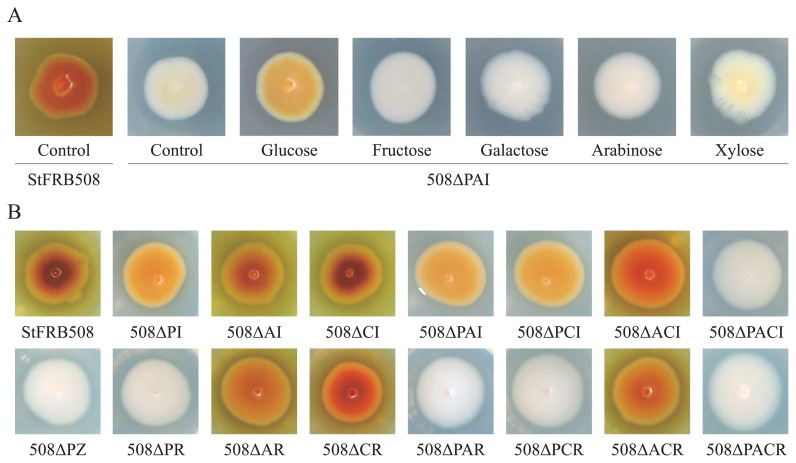
Phenazine production in StFRB508 and its mutants when grown on LB agar plates. (A) The 508ΔPAI mutant strain was inoculated on LB agar containing 1% (w/v) glucose, fructose, galactose, arabinose, and xylose. StFRB508 and 508ΔPAI were inoculated on LB agar without monosaccharides as a control. (B) A series of mutants of StFRB508 were inoculated on LB agar containing 1% (w/v) glucose. After an incubation at 30°C for 2 d, phenazine production was identified by the presence of orange pigments.

In order to elucidate the relationship between quorum sensing-related genes and phenazine production, we constructed multiple gene deletion mutants using a non-marker mutagenesis technique. Our previous findings revealed that phenazine production was not detected in the culture supernatant of the *phzI aurI* double mutant (13). In the present study, we examined phenazine production using the non-marker *phzI aurI* double mutant (508ΔPAI) on LB agar. Similar to our previous findings, no significant amount of phenazine production was observed in 508ΔPAI (Fig. 1A). With respect to growth conditions that favor phenazine production, Yuan *et al.* demonstrated that glucose supplementation enhanced phenazine production in *Pseudomonas* sp. M-18Q (26). In order to investigate the effects of monosaccharides on phenazine production by 508ΔPAI, glucose, fructose, galactose, arabinose, and xylose were added to LB agar at a concentration of 1% (w/v). 508ΔPAI showed low-level phenazine production on LB agar containing glucose, suggesting that the double mutation in *phzI* and *aurI* was not sufficient to abolish phenazine production. This effect was not observed with the addition of other monosaccharides (Fig. 1A).

We then examined phenazine production by all constructed mutants on LB agar containing glucose (Fig. 1B). In cases of AHL-synthase gene mutants, mutations in *aurI* and *csaI* did not reduce phenazine production. The mutation in *phzI* induced a slight reduction in phenazine production. The triple mutant of *phzI*, *aurI*, and *csaI* (508ΔPACI) did not produce phenazine, which was similar to 508ΔPZ as a negative control. The AHLs produced by PhzI were assumed to strongly stimulate phenazine production, whereas those produced by AurI and CsaI only yielded a slight stimulation. Although *Pseudomonas* sp. CMR12a and *P. chlororaphis* subsp. *aureofaciens* 30–84 have a second quorum-sensing system in addition to the *phzI*/*phzR* system, phenazine production was only regulated by the AHLs produced by PhzI (5, 27). This is the first study to show the cross-regulation of phenazine production by AHLs produced by the three independent AHL synthases. In the case of AHL-receptor gene mutants, mutations in *aurR* and *csaR* did not affect phenazine production. However, a single mutation in *phzR* completely diminished phenazine production by StFRB508. These results suggest that the expression of the phenazine biosynthesis gene cluster is regulated by the PhzR receptor alone. A previous study reported that the *csaI*/*csaR* quorum-sensing system regulates the cell surface properties of *P. chlororaphis* subsp. *aureofaciens* 30–84 (27). However, visual differences in colony morphologies were not confirmed between StFRB508 and mutants of the *aurI*/*aurR* or *csaI*/*csaR* quorum-sensing system.

### Identification of the structures of AHLs produced by StFRB508 and its mutants

**Fig. 2:**
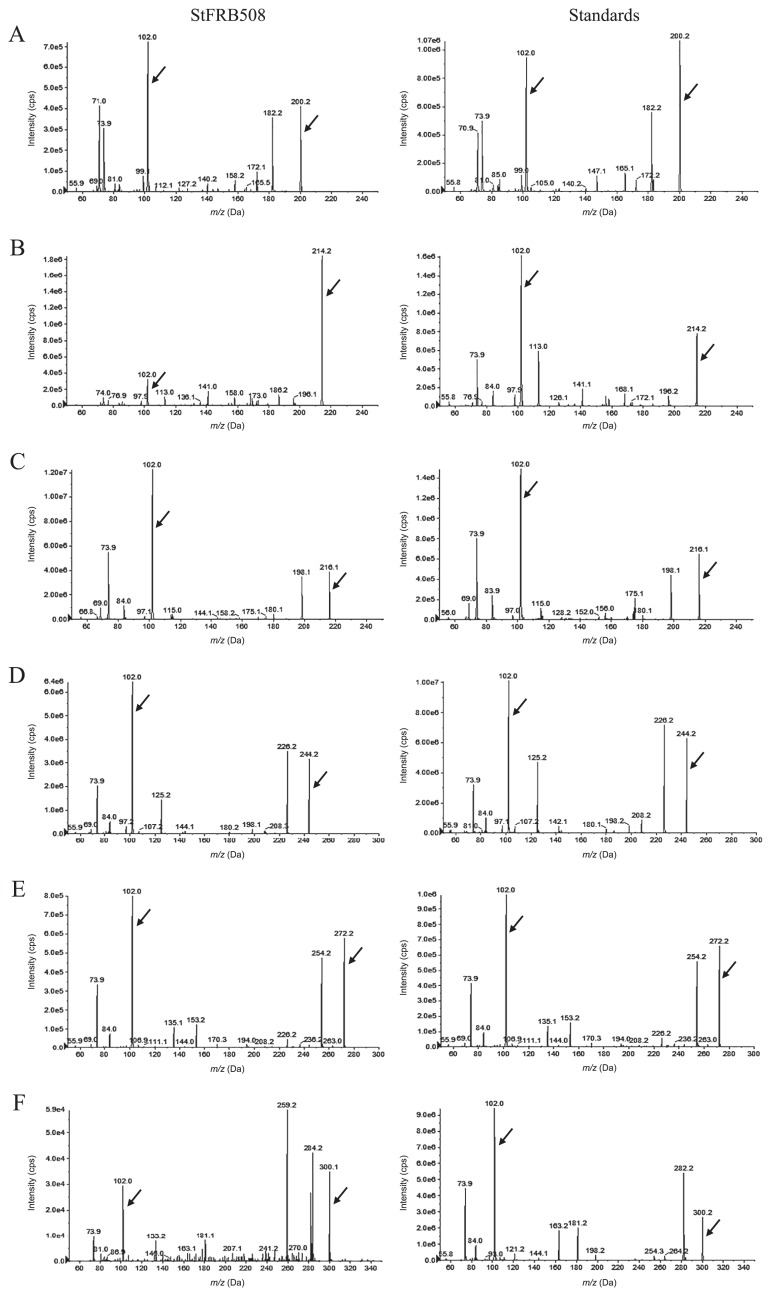
Mass spectra of AHLs extracted from the cell-free supernatant of StFRB508 (left panel) and AHL standards (right panel). After fractionation by reverse-phase HPLC, the ESI-MS/MS fragment peaks of AHLs were analyzed. All corresponding peaks for respective C6-HSL (A; *m*/*z* 200), 3-oxo-C6-HSL (B; *m*/*z* 214), 3-OH-C6-HSL (C; *m*/*z* 216), 3-OH-C8-HSL (D; *m*/*z* 244), 3-OH-C10-HSL (E; *m*/*z* 272), and 3-OH-C12-HSL (F; *m*/*z* 300) along with the product ion peaks (*m*/*z* 102) are marked by arrows.

We previously identified the structures of AHLs produced by PhzI and AurI using a TLC analysis combined with those produced by AHL-reporter strains (13). Our previous findings demonstrated that PhzI catalyzed the biosynthesis of 3-OH-C6-HSL, while AurI catalyzed that of C4-HSL and C6-HSL. However, the use of a TLC analysis to detect the structure of AHL is too simple, and difficulties are associated with elucidating specific structures or small amounts of AHLs. Therefore, in the present study, the chemical structures of AHLs extracted from StFRB508 and its mutants were analyzed by LC-MS/MS. The mass spectra of AHLs extracted from StFRB508 culture supernatants and AHL standards are shown in Fig. 2. Six AHLs—C6-HSL, 3-oxo-C6-HSL, 3-OH-C6-HSL, 3-OH-C8-HSL, 3-OH-C10-HSL, and 3-OH-C12-HSL—were detected in the StFRB508 culture supernatant. In order to elucidate the AHL structures produced by three AHL-synthase mutants, the culture supernatants of 508ΔACI (including the *phzI* gene only), 508ΔPCI (including the *aurI* gene only), and 508ΔPAI (including the *csaI* gene only) were prepared and assayed using LC-MS/MS. The culture extract of 508ΔACI contained four AHLs, 3-OH-C6-HSL, 3-OH-C8-HSL, 3-OH-C10-HSL, and 3-OHC12-HSL (Fig. S2). The culture supernatants of 508ΔPCI and 508ΔPAI contained two AHLs, C6-HSL and 3-oxo-C6-HSL (Fig. S3 and 4). We previously demonstrated using a TLC analysis that AurI produced C4-HSL instead of 3-oxo-C6-HSL (13). C4-HSL has also been detected in the culture supernatants of other *P. chlororaphis* strains using a TLC analysis (4, 10). Since the *R*_f_ values of C4-HSL and 3-oxo-C6-HSL were similar, we assumed that C4-HSL and 3-oxo-C6-HSL were not separated clearly by TLC. The *csaI*/*csaR* quorum-sensing system was reported for the first time from *P. chlororaphis* subsp. *aureofaciens* 30–84 (27). However, the structures of AHLs produced by CsaI were not elucidated in that study. As described above, CsaI from StFRB508 showed greater similarity to that from 30–84. Therefore, CsaI from *Pseudomonas chlororaphis* strains was assumed to commonly produce C6-HSL and 3-oxo-C6-HSL.

### Effects of AHL structures on phenazine production in AHL-deficient mutants

AHLs produced by wild-type StFRB508 were extracted after a 24-h cultivation and quantified by the LC-MS/MS system (Table 2). StFRB508 mainly produced 3-hydroxy-substituted AHLs, and 3-OH-C6-HSL showed the highest concentration (approximately 6 μM) among the six AHLs produced. The concentration and composition ratio of 3-hydroxy-substituted AHLs in 508ΔACI (for PhzI) were similar to those of wild-type StFRB508. In contrast, 508ΔPCI (for AurI) and 508ΔPAI (for CsaI) produced higher concentrations of C6-HSL and 3-oxo-C6-HSL than StFRB508. Since AHLs are produced by AHL synthase from *S*-adenosyl-l-methionine (SAM) and an acylated acyl carrier protein (16), the inactivation of PhzI caused an excessive supply of SAM toward AurI or CsaI in 508ΔPCI and 508ΔPAI. Although AurI and CsaI both produce C6-HSL and 3-oxo-C6-HSL, the composition ratio of the two AHLs is opposite in the products of AurI and CsaI.

**Fig. 3:**
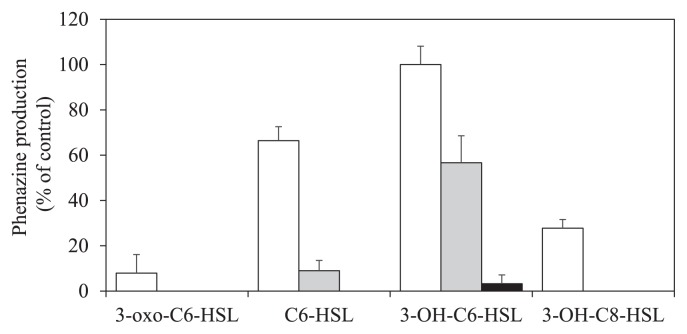
Phenazine production by 508ΔPACI in LB medium containing AHLs at concentrations of 0.1 (black bars), 1 (gray bars), and 10 μM (white bars). Relative phenazine production was evaluated using the value of A_365_/OD_600_; the maximum value obtained for 10 μM 3-OH-C6-HSL was set as 100%. Results were reproduced in three repeated experiments, and error bars indicate standard deviations.

In order to evaluate the effects of AHLs produced by StFRB508 on phenazine production, 508ΔPACI was inoculated into LB medium containing AHLs at a final concentration of 0.1, 1, or 10 μM. The results of the assay for phenazine production are shown in Fig. 3. 3-OH-C6-HSL exerted the strongest effects on phenazine production and also stimulated it at the lowest concentration tested (0.1 μM). Although C6-HSL induced a certain amount of phenazine production at a concentration of 1 μM, this induction level was 6-fold lower than that of 3-OH-C6-HSL. 3-oxo-C6-HSL and 3-OH-C8-HSL slightly induced phenazine production at a concentration of 10 μM. Neither 3-OH-C12-HSL nor 3-OH-C10-HSL stimulated phenazine production at the concentrations tested (data not shown). The null mutant of three AHL synthase genes, 508ΔPACI, produced a small amount of 3-OH-C10-HSL (Table 1). A complete genome analysis of StFRB508 revealed the presence of the hdtS gene homologue, which encodes a third class of AHL synthase. *E. coli* harboring *hdtS* from *P. fluorescens* F113 is capable of synthesizing three kinds of AHLs (9). The small amount of 3-OH-C10-HSL detected in the AHL extracts of 508ΔPACI was assumed to be produced by HdtS. However, since phenazine production was not stimulated in the presence of 3-OH-C10-HSL, the expression of the phenazine biosynthesis gene cluster may not be practically affected by 3-OH-C10-HSL.

### A quorum-sensing mutant exhibited decreased biocontrol efficacy

In our previous study, growth inhibitory activity against the phytopathogenic fungus, *F. oxysporum*, was attenuated in a phenazine-deficient mutant of StFRB508 in *in vitro* antagonism tests (13). In order to evaluate the contribution of AHL production to the *in vivo* biocontrol capacity of StFRB508, we used a cucumber-*P. ultimum* pathosystem. The addition of strain StFRB508 to pathogen-infested soil increased plant survival by approximately 3-fold, which confirmed this strain as an effective biocontrol agent (Table 2). In contrast, the AHL-deficient mutant, 508ΔPACI, had a significantly impaired capacity to protect cucumber from disease symptoms. In the case of *Pseudomonas* sp. CMR12a, the quorum-sensing mutant deficient in phenazine production had a significantly lower biocontrol capacity against *Rhizoctonia* root rot on bean plants than the wild-type (5). Our results also demonstrated that biocontrol activity through phenazine production was regulated by AHL-mediated quorum sensing in StFRB508. Although phenazine production is considered to be a major factor endowing this strain with plant protection efficacy, its relevance to other phenotypes such as bacterial motility and colonization needs to be established in future studies.

### Concluding Remarks

**Fig. 4:**
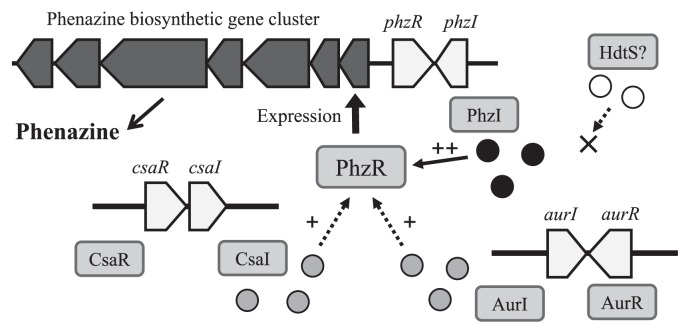
Schematic representation of the phenazine biosynthetic gene cluster by quorum sensing in StFRB508. Pentagons show the orientation and size of the genes. AHLs produced by PhzI (black circles) or AurI/CsaI (gray cycles), strongly (++) or weakly (+) bind to the PhzR receptor protein, respectively. The PhzR-AHL complex binds to the promoter and induces the expression of the phenazine biosynthetic gene. The putative AHL products by HdtS (open cycles) do not bind to the PhzR receptor.

Based on the present results, we propose a mechanism for the regulation of phenazine production by AHL-mediated quorum sensing in StFRB508 (Fig. 4). The results of the AHL quantification demonstrated that of all AHLs, 3-OH-C6-HSL, which exhibited the strongest activity for activating the phenazine biosynthesis gene cluster, was produced by PhzI at the highest concentration in StFRB508. In addition, 3-oxo-C6-HSL and C6-HSL, which were produced by AurI and CsaI, induced only the slight activation of the gene cluster in parallel with *phzI*/*phzR* system-mediated regulation. Although AurR and CsaR do not function as response regulators of the phenazine biosynthetic gene cluster, AHLs produced by AurI and CsaI exerted heterologous effects on the unrelated *phzI*/*phzR* quorum-sensing system. The true target genes of the *aurI*/*aurR* and *csaI*/*csaR* quorum-sensing systems have not yet been elucidated. Regarding phenazine production, unexpected spontaneous mutations in up to two AHL-synthase genes may not affect phenazine production. AHLs produced by AurI and CsaI may function as backup signals in the *phzI*/*phzR* quorum-sensing system in StFRB508.

## Supplementary Information



## Figures and Tables

**Table 1:** Suppression of *Pythium*-induced damping-off and root rot in cucumber by *Pseudomonas chlororaphis* subsp. *aurantiaca* StFRB508 and 508ΔPACI.

Bacterial strain added^a^	*Pythium* added^a^	Surviving plants per flask (%)^b^
None	−	100 a
StFRB508	−	100 a
508ΔPACI	−	100 a
None	+	21 c
StFRB508	+	63 b
508ΔPACI	+	25 c

a The *Pseudomonas* strain was added at 2×10^7^ CFU g^−1^ soil to100-mL flasks (50 mL of soil per flask), after planting three 48-h-old, sterile-grown cucumber seedlings per flask. *P. ultimum* was added as a millet-seed inoculum at 2.5 g kg^−1^ of vermiculite before planting. Plants were harvested after 7 d.

b Data represent the average of 10 replicates (flasks containing three cucumber plants) per treatment without *P. ultimum* and 16 replicates per treatment with *P. ultimum*. Means within the same column followed by different letters (a–c) are significantly different (*P*<0.05) according to Tukey’s HSD test.

**Table 2:** Quantification of AHLs produced by StFRB508 and its mutants.

Strains	StFRB508	508ΔACI	508ΔPCI	508ΔPAI	508ΔPACI

Functional AHL synthase	PhzI, AurI, CsaI	PhzI	AurI	CsaI	None
C6-HSL	218±43^a^	N.D.^b^	371±86	4177±28	N.D.
3-oxo-C6-HSL	159±67	N.D.	3205±692	426±235	N.D.
3-OH-C6-HSL	6006±713	6653±2506	N.D.	N.D.	N.D.
3-OH-C8-HSL	1282±412	1946±874	N.D.	N.D.	N.D.
3-OH-C10-HSL	768±210	809±413	N.D.	N.D.	8±12
3-OH-C12-HSL	2±0	3±1	N.D.	N.D.	N.D.

a AHL concentrations are expressed in nM.

b N.D., not detected.
