# Supplementary material for: Complete Genome Sequence of Pseudomonas chlororaphis subsp. aurantiaca Reveals a Triplicate Quorum-Sensing Mechanism for Regulation of Phenazine Production
Source: Microbes Environ. 2017 Feb 25;32(1):47–53. doi: 10.1264/jsme2.ME16162 (PMC5371074; doi:10.1264/jsme2.ME16162)
Supplement: Supplementary file 1 [file 32_47_s1.pdf]

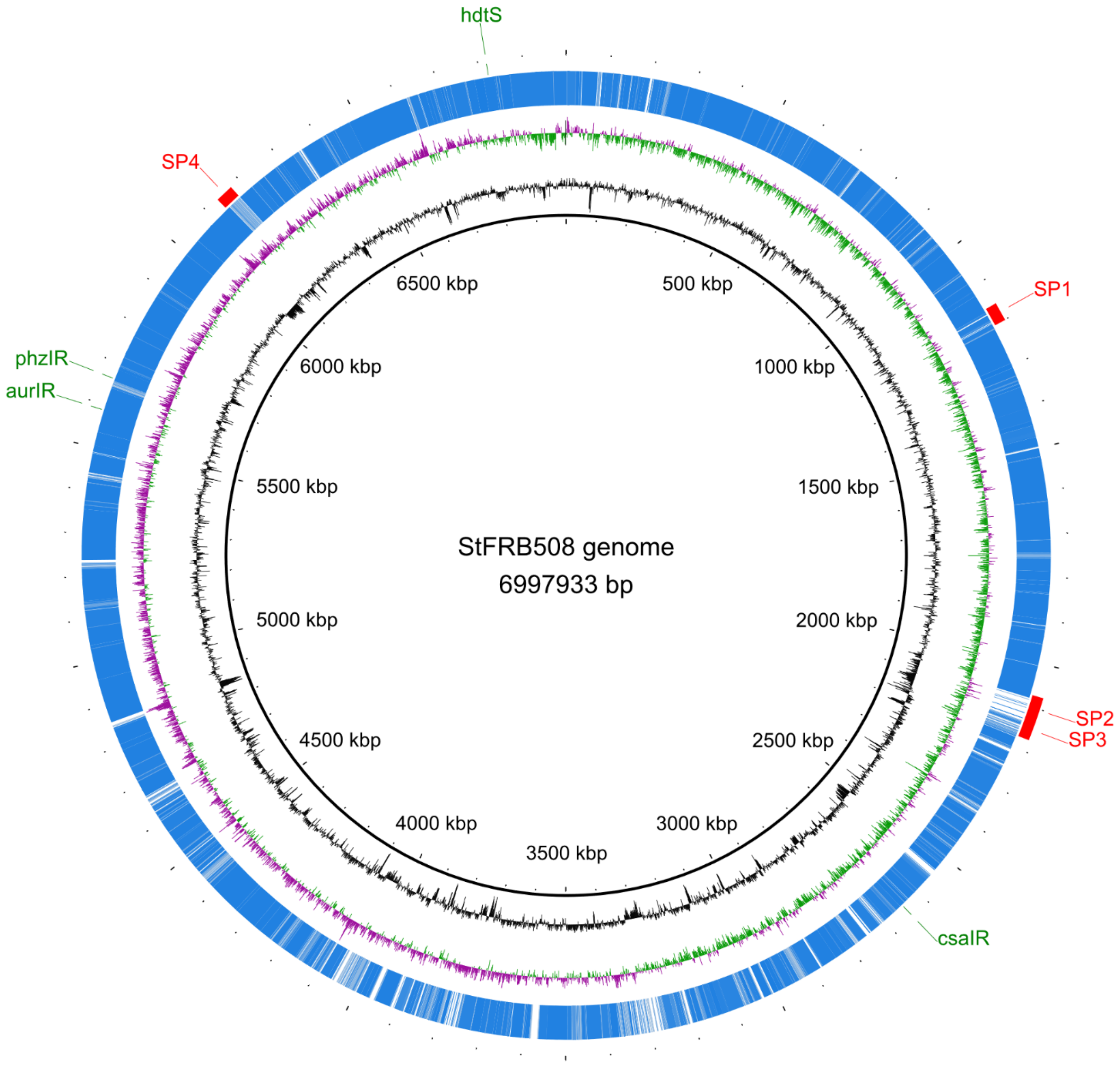

Fig S1. Genome map of StFRB508. Starting from the inside, circle 1 represents the mean of centered G + C content (bars facing outside, above mean; bars facing inside, below mean); circle 2 shows the GC skew (G – C)/(G + C); circle 3 represents comparison of gene content with *P. chlororaphis* subsp. *aurantiaca* JD37; the outermost ring highlights the regions of genetic elements. Prophage (SP) is shown in red and quorum sensing-related genes are shown in green. The inner black circle shows the scale line in kbps. The circular map that illustrates the general genomic feature as well as its comparison with another strain was plotted by using the BRIG program (http://brig.sourceforge.net/). The putative prophage regions were identified by PHAST program (http://phast.wishartlab.com/).

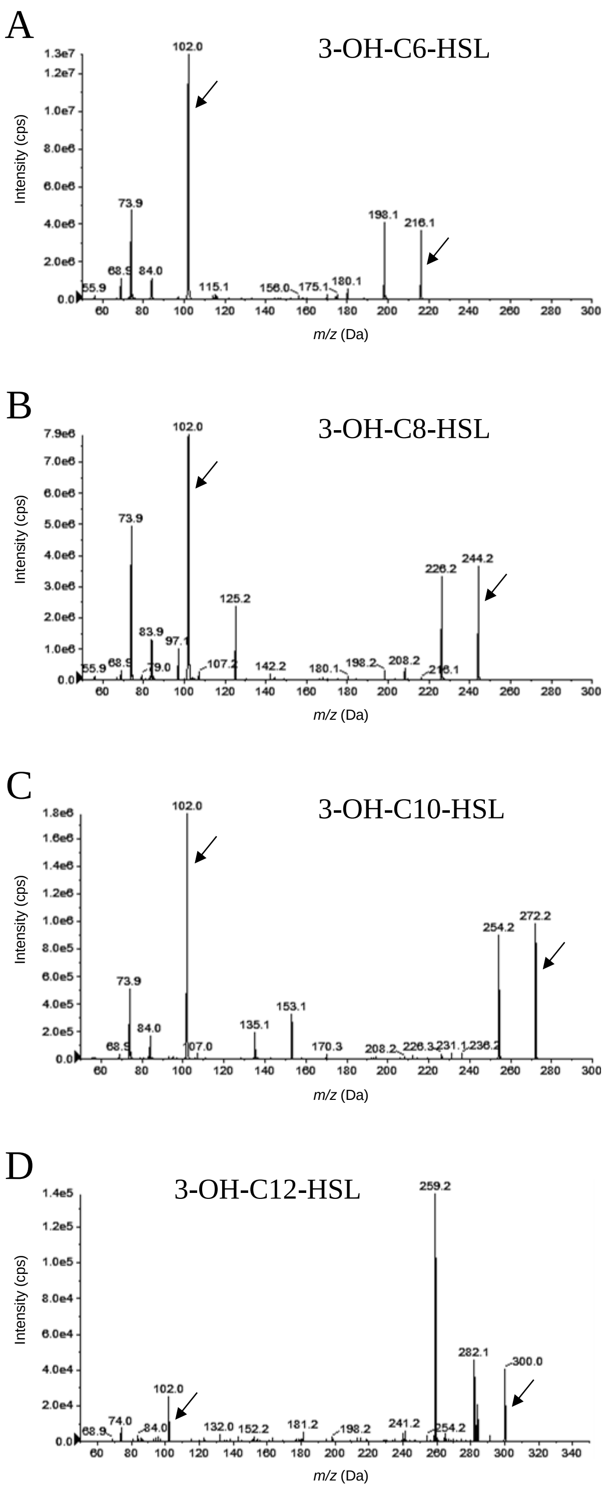

Fig. S2. Mass spectra of AHLs extracted from the cell-free supernatant of 508ACI for PhzI. After fractionation by reverse-phase HPLC, the ESI-MS/MS fragment peaks of AHLs were analyzed. All corresponding peaks for respective 3-OH-C6-HSL (A; *m/z* 216), 3-OH-C8-HSL (B; *m/z* 244), 3-OH-C10-HSL (C; *m/z* 272), and 3-OH-C12-HSL (D; *m/z* 300) along with the product ion peaks (m/z 102) are marked by arrows.

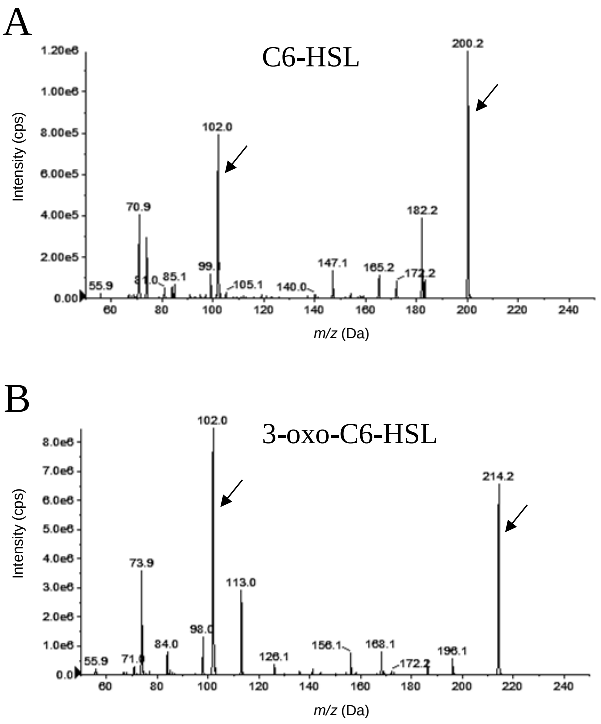

Fig. S3. Mass spectra of AHLs extracted from the cell-free supernatant of 508PCI for AurI. After fractionation by reverse-phase HPLC, the ESI-MS/MS fragment peaks of AHLs were analyzed. All corresponding peaks for respective C6-HSL (A; *m/z* 200) and 3-oxo-C6-HSL (B; *m/z* 214) along with the product ion peaks (*m/z* 102) are marked by arrows.

A

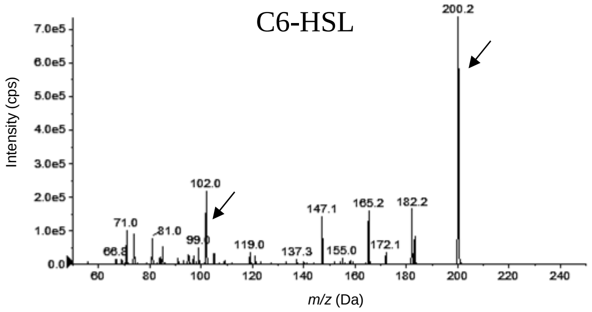

B

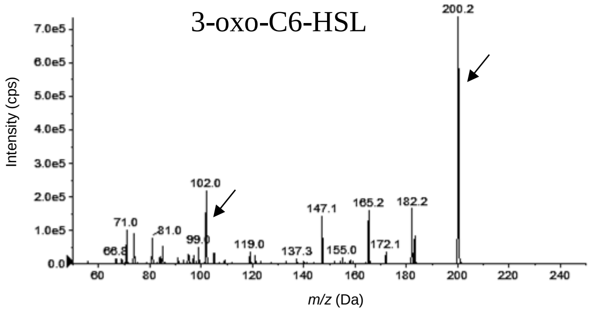

Fig. S4. Mass spectra of AHLs extracted from the cell-free supernatant of 508PAI for CsaI. After fractionation by reverse-phase HPLC, the ESI-MS/MS fragment peaks of AHLs were analyzed. All corresponding peaks for respective C6-HSL (A; *m/z* 200) and 3-oxo-C6-HSL (B; *m/z* 214) along with the product ion peaks (*m/z* 102) are marked by arrows.

Table S1. Bacterial strains used in the study

Strains	Description	Source
Escherichia coli		
DH5α	F^- *supE44* Δ*lacU169* (φ80 *lacZ*ΔM15) *hsdR17 recA1 endA1 gyrA96 thi-1 relA1*	Nippon Gene
S17-1 λ*pir*	*thi pro hsdR hsdM*$^+$ *recA* RP4 2Tc::Mu-Km::Tn7	19
Pseudomonas chlororaphis subsp. *aurantiaca*		
StFRB508	Phenazine and AHL producer isolated from potato root surface	11
508ΔPI	StFRB508 derivative, Δ*phzI*	This study
508ΔAI	StFRB508 derivative, Δ*aurI*	This study
508ΔCI	StFRB508 derivative, Δ*csaI*	This study
508ΔPAI	StFRB508 derivative, Δ*phzI* and Δ*aurI*	This study
508ΔACI	StFRB508 derivative, Δ*aurI* and Δ*csaI*	This study
508ΔPCI	StFRB508 derivative, Δ*phzI* and Δ*csaI*	This study
508ΔPACI	StFRB508 derivative, Δ*phzI,* Δ*aurI,* and Δ*csaI*	This study
508ΔPR	StFRB508 derivative, Δ*phzR*	This study
508ΔAR	StFRB508 derivative, Δ*aurR*	This study
508ΔCR	StFRB508 derivative, Δ*csaR*	This study
508ΔPAR	StFRB508 derivative, Δ*phzR* and Δ*aurR*	This study
508ΔACR	StFRB508 derivative, Δ*aurR* and Δ*csaR*	This study
508ΔPCR	StFRB508 derivative, Δ*phzR* and Δ*csaR*	This study
508ΔPACR	StFRB508 derivative, Δ*phzR*, Δ*aurR*, and Δ*csaR*	This study
508ΔPACR	StFRB508 derivative, Δ*phzR*, Δ*aurR*, and Δ*csaR*	This study
508ΔPZ	StFRB508 derivative, *phzCDE*::Km^r	11

Table S2. Primers used in the study

Description	Primers	Primer sequences (5'-3')*
Amplification of *phzIR*	phzIR-F	GCACTGGCGGCTACAGGAGGGAATTGGATAAACGG
	phzIR-R	GATCATTAAAGCCGCTAGGGGAAAGCGAAGCAGGC
Deletion of *phzI* internal sequence	phzIdel-F	TCTAAGCTTCCGACACATTATTGAAAGAGACAGC
	phzIdel-R	TCTAAGCTTCATCGCTCATTTCGTTCAGTGTGTGC
Deletion of *phzR* internal sequence	phzRdel-F	TCTAAGCTTCGACAGCCGTAAACTCCTGCATATCC
	phzRdel-R	TCTAAGCTTGGCAGTGTCTTACGCAGTCGCGATGG
Amplification of *aurIR*	aurIR-F	CGAGCAGATGGCGCCATAACCGGTTACAACTACCC
	aurIR-R	CAAGGTCTGGCAGGCGCTGCTGACCATTCCTTTCG
Deletion of *aurI* internal sequence	aurIdel-F	TCTAAGCTTGTGCTGTTGCTGAACATGCCGATCAG
	aurIdel-R	TCTAAGCTTGGCGTCGCACTATAGTCGAGCGTGTG
Deletion of *aurR* internal sequence	aurRdel-F	TCTAAGCTTGCCAGAGTATTTGAGTCGACTATCC
	aurRdel-R	TCTAAGCTTCATTTCCGGAAAGCTGCAGGTCTGC
Amplification of *csaIR*	csaIR-F	GCGCTTTCATATCGAACAGGCGCTCAGGCTCAACC
	csaIR-R	CCACCTGCTGATGGTGGCGATGGTGCTGTTCTTCG
Deletion of *csaI* internal sequence	csaIdel-F	TCTAAGCTTGCTTCGCTGAATCGGGTATTTCTGCG
	csaIdel-R	TCTAAGCTTGTGTCGGTGAAAGCTGGCTTTCATGC
Deletion of *csaR* internal sequence	csaRdel-F	TCTAAGCTTCTTCCACCTCAAGGTCATCCAGAAG
	csaRdel-R	TCTAAGCTTGGGAATCTAGTCCAGGGCAATACGC

**Hin*dIII sites are underlined.
